# Sustaining Mpox Surveillance, Research, and Care Integration in a Post-PHEIC, Resource-Constrained World

**DOI:** 10.3390/v18080844

**Published:** 2026-08-01

**Authors:** Patrick D. M. C. Katoto, Liliane Nsuli Byamungu

**Affiliations:** 1 Centre for Tropical Diseases and Global Health, Catholic University of Bukavu, Bukavu P.O. Box 285, Democratic Republic of the Congo; byamungu.nsuli@gmail.com; 2 Cochrane South Africa, South African Medical Research Council, Division of Epidemiology and Biostatistics, Department of Global Health, Faculty of Medicine and Health Sciences, Stellenbosch University, Cape Town 7505, South Africa; 3 Department of Paediatrics and Child Health, School of Clinical Medicine, College of Health Sciences, University of KwaZulu-Natal, Durban 4013, South Africa

**Keywords:** mpox, monkeypox, PHEIC, endemic disease management, HIV integration, sexually transmitted infections, Africa CDC, resource-constrained settings

## Abstract

In August 2024, the Africa Centres for Disease Control and Prevention (Africa CDC) declared mpox a Public Health Emergency of Continental Security (PHECS), and the World Health Organization (WHO) followed with a second Public Health Emergency of International Concern (PHEIC), in response to the rapid expansion of clade Ib monkeypox virus (MPXV) across eastern Democratic Republic of the Congo (DRC) and neighboring countries. Both declarations have since been lifted (September 2025 and January 2026), yet clade Ib transmission and severe outcomes in pregnant women and children persist. We argue that closing these emergency mechanisms marks not the end of the epidemic but the start of a harder phase: sustaining surveillance, care, and research amid shrinking donor support, including the dissolution of the United States Agency for International Development (USAID). We contend that integrating mpox into existing HIV, sexually transmitted infection (STI), and reproductive health platforms is the most realistic route to durable routine care, while pregnancy, paediatric disease, severe cases, and zoonotic spillover still need dedicated pathways. Sustainable management ultimately depends on domesticated financing, stronger institutions, genuine community engagement, and action on the ecological drivers of spillover.

## 1. Introduction

On 13 August 2024, the Africa Centres for Disease Control and Prevention (Africa CDC) declared mpox a Public Health Emergency of Continental Security (PHECS), the first such declaration in the continent’s history, in response to escalating clade-Ib monkeypox virus (MPXV) transmission spreading from eastern Democratic Republic of the Congo (DRC) into Uganda, Rwanda, Burundi, and beyond [1]. One day later, the World Health Organization (WHO) announced a second mpox Public Health Emergency of International Concern (PHEIC) [2]. Together, the declarations reframed mpox: no longer primarily the sexually transmitted outbreak described in 2022 [3], but an evolving endemic disease of African origin, marked by new transmission dynamics, expanding geography, and severe outcomes in pregnant women and children.

The scale of what followed these declarations warrants a brief overview. Africa CDC recorded 80,276 suspected cases and 1340 deaths across the continent in 2024 alone, more than a five-fold rise in cases and a two-fold rise in deaths relative to the equivalent period of 2023, with the DRC accounting for 96% of reported cases and 97% of deaths [4]. Between 1 January 2025 and 14 June 2026, 31 African countries reported 49,176 laboratory-confirmed cases and 222 deaths among confirmed cases (case fatality ratio 0.5%), against a far higher fatality ratio among suspected cases in settings where confirmatory testing is limited [5]. The continental response mobilised more than US$1 billion, expanded laboratory and genomic sequencing capacity more than ten-fold, and deployed over five million vaccine doses across 16 countries; between peak transmission in early 2025 and late 2025, suspected cases fell by 40% and confirmed cases by 60%, while the case fatality ratio among suspected cases declined from 2.6% to 0.6% [4]. These gains are substantial and were achieved largely through African leadership. They were also achieved through emergency financing, and transmission has not ceased: clade Ib was newly introduced into Madagascar in December 2025 and accounted for the majority of confirmed cases reported on the continent during the first half of 2026 [5].

A brief virological note may assist the non-specialist reader. MPXV is a large double-stranded DNA virus of the genus Orthopoxvirus, family Poxviridae, closely related to variola virus and sufficiently cross-reactive with vaccinia virus that vaccinia-based vaccines confer protection against mpox. Two genetically distinct clades are recognised. Clade I, historically associated with the Congo Basin, comprises clade Ia, which is maintained largely by repeated zoonotic introduction from forest reservoirs, and clade Ib, which emerged recently; clade I has consistently been associated with higher case fatality and with severe disease in children, pregnant women, and immunocompromised people. Clade II, associated with West Africa, comprises clade IIa and clade IIb, the latter responsible for the 2022 global outbreak that spread principally through sexual networks among men who have sex with men and carried comparatively low mortality. Clade Ib, first characterised in South Kivu in 2023, carries APOBEC3-type mutational signatures indicating sustained replication in humans rather than recent animal origin, and, unlike clade Ia, transmits efficiently through sexual and close-contact networks without requiring repeated spillover [6,7,8]. Throughout this Commentary, we use MPXV to denote the virus and mpox to denote the disease it causes, following current WHO nomenclature.

The clade Ib outbreak, first detected in Kamituga, South Kivu, in late 2023, demonstrated efficient, largely heterosexual transmission within mining-associated commercial sex networks [9]. It spread through South Kivu into urban centres such as Bukavu and Kinshasa, into multiple DRC provinces, and across international borders along human mobility corridors [6,7,10]. Severe outcomes, including foetal loss rates approaching 50% and disproportionately high paediatric mortality, distinguished clade-Ib from the largely mild clade IIb outbreak of 2022 [11].

This convergence of transmissibility, geographic spread, and clinical severity defines the central challenge of what we call the post-PHEIC era: sustaining response capacity after emergency mechanisms dissolve. The challenge is compounded by a deteriorating financing landscape, including the dissolution of the United States Agency for International Development (USAID), which ceased implementing foreign assistance on 1 July 2025 with residual functions absorbed into the U.S. Department of State, which has opened critical gaps precisely where the clade I burden is heaviest [12]. In this Commentary, we argue that integration into HIV, sexually transmitted infection (STI), and reproductive health platforms is the most viable route to sustainable mpox management at scale, and we outline the institutional, financing, and research priorities needed to support it.

## 2. The Post-PHEIC Paradox

The PHEIC mechanism is intrinsically temporary, designed to trigger global mobilization rather than to sustain long-term disease control. Closure of a PHEIC often reflects reduced risk to high-income countries rather than control achieved in endemic countries themselves.

The 2023 closure of the mpox PHEIC largely reflected declining clade IIb activity in Europe and North America, not control of clade I transmission in Central Africa [13]. In fact, the period immediately following that closure saw the early expansion of clade Ib that would later force a second PHEIC and Africa’s first PHECS declaration [1,2]. African scientists had raised concerns throughout this period, but global attention shifted only once international spread became visible. The sequence has now repeated. The WHO lifted the second mpox PHEIC on 5 September 2025 [14], and Africa CDC lifted the PHECS on 22 January 2026 [4], both citing sustained declines in reported cases. Those declines are real, but they coincided with a documented contraction in surveillance activity that the WHO itself cautions may cause cases to be underestimated, and with continuing introductions into countries previously unaffected [5]. The epidemiological signal that ends an emergency is therefore partly a function of how hard the system is still looking.

This “post-PHEIC paradox,” in which formal resolution coincides with a worsening endemic burden, is not unique to mpox; it has recurred after Ebola outbreaks and COVID-19 emergency transitions [15], reflecting a structural misalignment between global emergency mechanisms and the sustained burdens borne by low- and middle-income endemic settings [16]. In practice, the end of a PHEIC tends to bring the closure of emergency procurement channels, the discontinuation of surge-funded laboratory support, the withdrawal of emergency health workforce deployments, declining partner attention and financing, reduced vaccine donations, and a degradation of surveillance reporting. For endemic communities, there is no “end” to mpox, only the end of emergency support. Sustainable management therefore requires reframing mpox as an endemic disease that warrants the same permanent institutional commitment as HIV, tuberculosis, or malaria, rather than a recurring emergency to be managed episodically.

## 3. Financing the Transition

The USAID has long been a major funder of HIV programming (via the U.S. President’s Emergency Plan for AIDS Relief, PEPFAR), maternal and child health, laboratory systems, and infectious disease surveillance across sub-Saharan Africa; its dissolution therefore has profound implications for the mpox response [12]. The most immediate consequences include instability in HIV programmes (the strongest existing platform for mpox integration), threatened laboratory functionality (including consumables for MPXV polymerase chain reaction (PCR) testing), attrition among community health workers partly funded through USAID mechanisms, and weakening of the surveillance platforms that generate routine case data. Many “emergency-period” mpox investments were never fully incorporated into national budgets and were therefore especially vulnerable to this donor contraction [10,12].

Beyond seeking alternative donors, mpox programmes must progressively shift toward domestic financing: embedding mpox-related costs into existing HIV, STI, reproductive health, and immunization budget lines; avoiding vertical mpox programmes that require stand-alone management structures; incorporating mpox diagnosis and care into national health insurance benefit packages; and leveraging Africa CDC-coordinated pooled procurement to reduce commodity costs. Domestic financing is undeniably difficult under fiscal constraint, but donor-dependent surveillance and laboratory systems are inherently fragile.

Research is often the first casualty of post-emergency contraction, yet it remains essential for evidence-based endemic management. Longitudinal cohorts, clinical trials, and implementation science all require sustained, multi-year financing. Viable sources include national research councils, African research initiatives, and multilateral funders such as the European and Developing Countries Clinical Trials Partnership (EDCTP), the Fogarty International Center, and Wellcome; African-led consortia with established community infrastructure should be prioritized to build lasting local scientific capacity.

## 4. The Case for HIV and STI Platform Integration

Of all the institutional options available, integration into HIV and STI platforms is the most epidemiologically and operationally coherent model for routine mpox care. The risk groups overlap substantially, including sex workers, mobile mining populations, and individuals with multiple partners [17], and mpox severity is markedly higher among people living with HIV [18]. Clinical similarities with STIs enable diagnostic synergy, and HIV/STI clinics already possess much of the infrastructure mpox programmes need: trained staff, supply chains, laboratory linkages, and data systems that integration can leverage for cost-efficiency and scale [19].

In practice, integration means screening for mpox symptoms during routine HIV or STI clinic triage; training clinicians in mpox recognition and differential diagnosis, particularly distinguishing mpox from other vesicular and exanthematous conditions encountered in HIV/STI settings, including varicella-zoster virus and herpes simplex virus infections; ensuring PCR diagnostic capacity or clear referral pathways; establishing isolation and treatment protocols; and co-delivering mpox vaccination during pre-exposure prophylaxis (PrEP) visits. On the surveillance side, it means adding mpox to national notifiable-condition frameworks, incorporating mpox fields into electronic HIV/STI records, establishing sentinel mpox surveillance in HIV, STI, and antenatal settings, and ensuring interoperability across health information systems [20]. Community health workers, who support case identification, contact tracing, vaccination, and education, are pivotal to this model; extending their scope within existing HIV and reproductive health systems is both cost-effective and contextually appropriate [21].

Integration, however, is not a universal solution. Dedicated mpox pathways remain necessary for pregnant women, children, severe or complicated cases requiring hospitalization, and zoonotic spillover investigations. The pragmatic model is therefore one of system-level integration paired with specialized clinical pathways where the evidence demands them.

The cost implications of integration deserve explicit attention, since they are more often assumed than measured. The central economic argument for integration is that its dominant cost categories—clinical space, staff time, supply chains, laboratory linkages, and health information systems—are already financed within HIV and STI programmes, so the marginal cost of adding mpox is considerably lower than that of establishing a vertical programme. The genuine incremental costs are concentrated in a small number of items: PCR reagents, cartridges, and specimen transport; clinician and community health worker training; isolation capacity and personal protective equipment; vaccine doses and any cold-chain extension required to reach them; and the modest data-system modifications needed to add mpox fields to existing registers. Several of these can be reduced further. Multiplex and open polyvalent PCR platforms already deployed for HIV viral load, tuberculosis, and human papillomavirus testing can accommodate MPXV assays at a lower marginal cost per test than dedicated instruments, and Africa CDC-coordinated pooled procurement can reduce unit commodity prices. Offsetting savings arise from avoided duplication of triage, counselling, contact tracing, and clinic infrastructure. Against this, integration imposes real opportunity costs on already overstretched HIV and STI services, and these are borne by the same staff whose posts are most exposed to donor contraction. We are not aware of any published economic evaluation of integrated mpox-HIV/STI service delivery in a sub-Saharan African setting; costing and cost-effectiveness analysis is therefore included among the implementation research priorities set out in Section 6.

## 5. Institutional Architecture for the Routine Era

Sustaining mpox functions beyond the PHEIC requires clarity about which institutions hold which mandates (Table 1). National Institutes of Public Health, or their functional equivalents, serve as the technical backbone of surveillance and data governance, maintaining the national case registry, coordinating sentinel surveillance, leading outbreak investigations, updating clinical and laboratory guidelines, and liaising with the Africa CDC, WHO, and research partners, yet many remain under-resourced in epidemiology staff, digital infrastructure, and sequencing capacity.

The Expanded Programme on Immunization (EPI) holds the cold-chain systems and workforce needed to deliver mpox vaccination at scale. Its role in the endemic era shifts from emergency mass campaigns to risk-stratified vaccination targeting people living with HIV (especially with CD4 counts below 350 cells/µL), sex workers and individuals in high-transmission networks, healthcare workers in high-risk settings, household contacts, and pregnant women with suspected exposure. Three vaccine platforms are relevant to this task, and the differences between them carry operational consequences. MVA-BN (marketed as JYNNEOS, IMVANEX, or IMVAMUNE) is a third-generation, replication-deficient modified vaccinia Ankara vaccine given as two subcutaneous doses 28 days apart; it is the platform on which the African response has principally relied, with more than five million doses deployed across 16 countries [4]. Its replication incompetence makes it appropriate for immunocompromised recipients, including people living with HIV, and its safety has been assessed across more than twenty clinical trials without safety signals in people with HIV, atopic dermatitis, children, or pregnant and breastfeeding women, although sample sizes in these subgroups remain small [22]. Real-world effectiveness estimates for two doses given as pre-exposure prophylaxis during the clade IIb outbreak fall broadly in the range of 66–90%, with clade I-specific effectiveness data still limited and duration of protection incompletely characterised [22,23]. LC16m8 is a minimally replicating third-generation vaccine with the largest paediatric safety experience, including approximately 50,000 children, administered as a single percutaneous dose; it remains contraindicated in pregnancy and severe immunosuppression and has limited effectiveness data from outbreak settings [23]. ACAM2000 is a second-generation, fully replication-competent vaccinia vaccine associated with myocarditis, pericarditis, progressive vaccinia, and autoinoculation, contraindicated in pregnancy and immunosuppression, and not licensed for mpox in most jurisdictions [23]. Because the populations at greatest risk in this epidemic include people living with HIV, pregnant women, and young children, MVA-BN remains the platform of choice in most African settings, with LC16m8 a defensible alternative where paediatric coverage is the priority and immunosuppression can be excluded. EPI also carries pharmacovigilance responsibilities for populations under-represented in pre-licensure data, while procurement barriers, intellectual property constraints, and cold-chain limitations continue to restrict equitable vaccine access [24,25]. The decline in orthopoxvirus immunity since the cessation of routine smallpox vaccination further justifies proactive mpox vaccination strategies [26], with transmission dynamics and genomic data informing deployment prioritization [7].

National Reference Laboratories anchor diagnostic confirmation, genomic sequencing, and external quality assurance, but routine management demands decentralized PCR capacity at regional laboratories: turnaround delays of several weeks, common when peripheral sites depend solely on a national reference laboratory, are incompatible with clinical decision-making and surveillance needs. The WHO, for its part, provides essential normative functions, including case definitions, International Health Regulations (IHR) reporting standards, Strategic Advisory Group of Experts (SAGE)-led vaccine policy, global stockpile access, and laboratory quality frameworks [16], but this normative guidance must be paired with implementation-focused technical support embedded in WHO country offices if it is to translate into functional national programmes.

One set of mandates was largely absent from the emergency-era architecture and must be built into the routine one: animal and environmental health. Mpox is a zoonosis, and no arrangement that assigns responsibility solely to human-health institutions can address the reservoir dynamics that generate new introductions. National veterinary services, wildlife authorities, and environment and forestry ministries hold the mandates for reservoir and wildlife surveillance, investigation of suspected spillover events, oversight of wildlife trade and bushmeat markets, and monitoring of land-use change at forest margins; in most affected countries these bodies are neither funded for mpox nor formally linked to human mpox surveillance. Operationalising this requires designated One Health coordination mechanisms with standing membership from the human, animal, and environmental sectors; joint outbreak investigation protocols that trigger animal-side sampling when a human case has no identifiable human source; shared laboratory capacity, since the same PCR and sequencing platforms serve human and animal specimens; and data-sharing arrangements that permit genomic comparison across sectors. Ministries of education and local government have a complementary role through school- and community-based hygiene and safe-handling education in hunting and forest-margin communities, and through the land-use and settlement planning that governs how rapidly forest frontiers are converted. These are inexpensive additions relative to their preventive value, and they are the mechanisms by which mpox control moves upstream of the clinic (Table 1).

## 6. A Research Agenda and Special Population Priorities

Four research priorities should anchor the routine era. First, continuous genomic surveillance, meaning sequencing at least 10% of confirmed cases with standardized protocols and timely repository uploads, is essential to detect clade evolution, lineage introductions, and geographic connections; the African Pathogen Genomics Initiative provides a continental coordinating structure [27]. Second, longitudinal cohorts are needed to resolve major knowledge gaps around post-infection immunity duration, household attack rates, long-term sequelae, and reinfection risk, ideally nested within existing HIV or reproductive health cohorts. Third, implementation science, addressing vaccine-uptake barriers among sex workers and mining communities, context-specific stigma, optimal community health worker training, and the cost-effectiveness of integrated models, should be embedded in operational research funding rather than treated as optional. Fourth, clinical and therapeutic research remains thin: tecovirimat showed no significant improvement in time to lesion resolution for clade I mpox in the PALM 007 trial [28], supportive care practices such as wound management, pain control, and nutrition remain severely understudied despite their clinical impact [29], and One Health surveillance at the animal-human interface, covering rodent reservoirs, land-use change, and wildlife trade, needs systematic investment given the continued risk of zoonotic spillover [8,30].

The One Health dimension warrants more than the passing mention it usually receives, because it bears directly on whether sustainable management is achievable at all. MPXV is enzootic in Central and West African forest ecosystems. Reservoir competence has not been definitively established for any single species, but the evidence points to widely distributed forest-dwelling rodents and squirrels; a 2023 outbreak in wild sooty mangabeys in Côte d’Ivoire was traced to a fire-footed rope squirrel carrying a genomically identical virus, the clearest demonstration to date of a sylvatic transmission chain [8]. The drivers of spillover are anthropogenic and lie largely upstream of the health sector: population growth in forested regions, deforestation and habitat encroachment, the expansion of artisanal mining and its associated settlements, reliance on wild animal protein in food-insecure areas, wildlife trade, and the waning of orthopoxvirus immunity in cohorts born after routine smallpox vaccination ceased [8,26]. This matters for the argument we are making. Clade Ib now spreads efficiently between humans and is, in the near term, amenable to the clinical and surveillance measures described above; but clade Ia continues to be seeded by repeated zoonotic introduction, and each introduction is an opportunity for a new human-adapted lineage to emerge, precisely as occurred in South Kivu. A response that acts only on human-to-human transmission is therefore permanently reactive, and permanently dependent on the emergency financing that follows each new lineage. Acting on the ecological drivers—through land-use planning that constrains unplanned forest conversion, regulation of and risk communication around wildlife trade and bushmeat handling, incorporation of mining settlements into environmental and health planning, and sustained hygiene education in forest-margin communities—is what converts mpox from a recurring emergency into a managed endemic problem. This is necessarily cross-sectoral work involving agriculture, environment, mining, education, and local government alongside health, and it should be resourced as such rather than deferred as a research aspiration.

Three populations require dedicated attention within this agenda. Pregnant women and neonates face foetal loss rates of roughly 50% and substantial maternal and neonatal morbidity in clade I and Ib infection [17,31], yet antenatal mpox screening is rarely implemented; closing that gap requires routine symptom inquiry, clinician training in pregnancy-specific presentation, and national guidelines on antiviral and vaccine use in pregnancy [32]. Children carry a disproportionate burden that is often under-detected: in the South Kivu cohort, 14% of cases occurred in children under five, and all reported deaths were in this age group [31]; integrating mpox into fever-rash protocols, training community health workers in paediatric recognition, and conducting dedicated antiviral dosing studies are priorities. Healthcare workers, finally, face elevated occupational risk and serve as a sentinel indicator of uncontrolled community transmission, warranting infection-prevention-and-control training, pre-exposure vaccination, occupational reporting pathways, and paid sick leave to support isolation.

## 7. Community Engagement and Foreseeable Challenges

Community engagement is not a peripheral add-on; it is foundational to case detection, stigma reduction, contact disclosure, vaccine uptake, and overall programme acceptability. Community health workers and civil society actors provided critical early detection in eastern DRC despite minimal support [33], and stigma, particularly where mpox is associated with commercial sex work, continues to undermine care-seeking and contact tracing. Effective engagement requires funding community groups as implementing partners, incorporating community representatives into national technical bodies, and developing non-stigmatizing clinical entry points tailored to key populations; it cannot remain optional or donor-dependent.

Programme design must also anticipate four structural challenges. Integration fatigue is real: frontline health workers and data systems are already overextended, so integration must streamline processes by using shared registers and harmonized data fields, rather than adding parallel workflows. Stigma and criminalization linked to commercial sex work amplify legal and social vulnerability, and community-led organizations often navigate these challenges more effectively than formal systems. Supply chain fragility, including reagent shortages, cold-chain failures, and procurement delays, undermined diagnostic and vaccination efforts during the clade Ib outbreak and calls for regional pooled procurement, buffer stocks, and manufacturer diversification. Finally, political prioritization remains precarious: mpox must compete with numerous urgent health priorities, and advocacy should frame its control not as a marginal infection but as a health security investment with direct implications for maternal, child, and community health.

## 8. Conclusions

The closure of the 2024 mpox emergency declarations did not end the epidemic; it ended only the emergency machinery built to respond to it. What remains is a now-entrenched endemic disease that will test the durability of African health systems for years to come. We have argued that integration into HIV, STI, and reproductive health platforms offers the most realistic, scalable foundation for routine mpox care [34], while pregnant women, children, severe disease, and zoonotic spillover continue to require dedicated pathways alongside that integrated backbone. We have argued equally that integration is not sufficient on its own. Without parallel investment in the animal and environmental health mandates that govern spillover, and without cross-sectoral action on deforestation, wildlife trade, land-use change, and community hygiene education, mpox control will remain a sequence of emergencies rather than a managed endemic programme, because the reservoir will keep generating the next lineage. None of this is achievable through donor goodwill alone. Domesticated financing, strengthened national public health institutions, and genuine, funded community engagement are structural prerequisites, not optional extras, for governing mpox, and the next entrenched pathogen, as the endemic disease it has become.

## Figures and Tables

**Table 1:** Institutional roles and core mpox mandates in the post-PHEIC era.

Institution/Body	Core Mpox Mandate in the Routine Era
National Institute of Public Health (NIPH)	National case registry; epidemiological and genomic surveillance; outbreak investigation; guideline oversight
Expanded Programme on Immunization (EPI)	Risk-group vaccination; cold chain management; AEFI monitoring; vaccine safety surveillance
National Reference Laboratory (NRL)	Confirmatory PCR; sequencing; EQA oversight; specimen transport; reagent stockpiling
National HIV/STI Programmes	Integrated screening and management; mpox-STI co-management; linked registers; PrEP-vaccine co-delivery
Reproductive & Maternal Health Programmes	Antenatal screening; perinatal management; neonatal surveillance; stillbirth attribution
Africa CDC	Continental surveillance; cross-border alerts; regional vaccine procurement; genomic coordination
WHO AFRO	Normative guidance; IHR standards; laboratory accreditation; global reporting; stockpile access
Community Organizations	Community-based detection; stigma reduction; vaccine uptake support; accountability and advocacy
National Veterinary Services, Wildlife and Environmental Health Authorities	Reservoir and wildlife surveillance; investigation of suspected spillover events; oversight of wildlife trade and bushmeat markets; monitoring of land-use change at forest margins; joint One Health coordination with human-health sectors
Ministry of Finance and National Health Insurance Scheme	Incorporation of mpox costs into domestic budget lines and insurance benefit packages; transition planning away from donor-dependent financing; sustained funding of community organizations as implementing partners

AEFI, adverse events following immunization; EQA, external quality assurance; IHR, International Health Regulations; PCR, polymerase chain reaction; PrEP, pre-exposure prophylaxis; STI, sexually transmitted infection. Institutions are listed in the order discussed in the text; mandates are indicative rather than exhaustive and will require adaptation to national administrative structures.

## Data Availability

No new data were created or analysed in this study. Data sharing is not applicable to this article.
